# Gains beyond cosmesis: Recovery of fusion and stereopsis in adults with longstanding strabismus following successful surgical realignment

**DOI:** 10.4103/0301-4738.60079

**Published:** 2010

**Authors:** K S Santhan Gopal

**Affiliations:** Kamalanethralaya, #81, 7^th^ cross, Koramangala, 4^th^ Block, Bangalore - 560 034, India

Dear Editor,

I read with interest the article by Fatima *et al*.,[1] It's well established that surgical alignment of the eyes in adults can result in recovery of binocular vision, even in cases with tropia and/or amblyopia.[2]

Not all tropias similar in size, direction and duration, have a similar postoperative course.

The patients included in the present study had good vision in both eyes indicating that the strabismus manifested after the completion of the critical period of binocular development. The actual age of the patient, at the time of onset of strabismus is not mentioned. The authors considered the children (six of them) in the age group of 12-16 as adults! This invalidates the conclusion of this study as, after strabismus surgery, the adults and children respond differently.

One must understand that, for recovery of binocularity, postoperatively, the age of the patient at the time of onset of strabismus as well as the total duration of strabismus are important. For example, a nine-month-old child with strabismus of just a few weeks' duration may have significant loss of binocularity, whereas a child with onset of strabismus at the age of four years may not have any loss of binocularity in a few months.

Adults with cataract may have sensory exotropia, due to disruption of the fusion. The cataract surgery with intraocular implant will restore binocularity and correct strabismus. In these patients preoperative evaluation may reveal no binocularity. Postoperatively many of these patients recover good binocular function and straight eyes.

What matters for recovery of binocular vision, is the age at which the strabismus started, rather than just the duration of the strabismus. The younger the age at onset of strabismus, the more disrupted will be the binocularity. In the current study, most patients have had exotropia (12) and few esotropia. Esotropias starting at an early age tend to disrupt binocular vision much more than exotropias with an onset at the same age. Tropia not reducing the vision in either of the eyes is unlikely to significantly affect the cortical binocular circuitry. Hence it is not unusual for these patients to have regained good binocularity postoperatively. Therefore, strabismus surgery for adults should not be denied on the wrong assumption of “poor postoperative functional recovery”, in longstanding strabismus.

The author's conclusion that adults do recover good binocularity after surgical correction of squint is true, but the approach route is incorrect! They have included children in the study and drawn conclusions for adults. Most patients in the study have a greater chance of recovery of binocularity postoperatively, due to age or the type of strabismus.
